# Evaluation of a national programme to improve shared decision-making skills among junior medical doctors in Denmark: a mixed methods study of satisfaction, usefulness, and dissemination of learning outcomes in clinical practice

**DOI:** 10.1186/s12913-022-07639-6

**Published:** 2022-02-23

**Authors:** Maria Helene Jacobsen, Cecilie Sommer, Siw Anna Wernberg, Helga Schultz, Sofie Charlotte Fage Hjortø, Maria Kristiansen

**Affiliations:** 1 Present Address: Center for Healthy Aging & Department of Public Health, Faculty of Health and Medical Sciences, University of Copenhagen, Copenhagen K, Denmark; 2 Present Address: Department of Oncology and Palliation, North Zealand Hospital, Frederikssund, Denmark; 3 Present Address: Department of Gynaecology and Obstetrics, Zealand University Hospital, Roskilde, Denmark

**Keywords:** Shared decision-making, Patient-centred care, SDM training programme, Mixed methods study, Evaluation

## Abstract

**Background:**

Shared decision-making (SDM) is a cornerstone in patient-centred care and there has been an increase in programmes aiming to improve clinicians’ abilities to engage in it. However, the evidence for such programmes’ effectiveness on clinicians’ use of SDM in clinical practice is sparse. The SDM Ambassador course, developed and facilitated by the Danish Association of Junior Doctors in Denmark (Junior Doctors Denmark) is a Danish SDM training programme for junior medical doctors (JMDs). This study aims to evaluate the SDM Ambassador course, with a focus on satisfaction, usefulness, and dissemination of learning outcomes in clinical practice.

**Methods:**

This is a mixed methods study, consisting of an online survey followed by semi-structured interviews. The participants were JMDs who had trained to be SDM ambassadors between May 2016 and September 2020 (n=185). The ambassadors were invited to participate in the survey and 112 ambassadors completed it, corresponding to a response rate of 61%. Descriptive statistics and χ^2^-tests were conducted. Subsequently, purposive sampling was used to identify 10 ambassadors for interviews. The interviews were transcribed, encoded, and subsequently analysed thematically. Finally, the quantitative and qualitative results were integrated.

**Results:**

Overall, the ambassadors were satisfied with their learning outcomes and experienced a greater capacity to unfold the perspectives of their patients. A majority (79%) reported that they had used SDM in their clinical practice with patients, and 59% had disseminated SDM to their colleagues. The usefulness and dissemination of learning outcomes in the clinic were shaped by the ambassadors’ perceptions of their moderate professional experience as junior doctors, and constrained by structural and cultural conditions in the context of their clinical practice.

**Conclusions:**

Despite overall satisfaction with their learning outcomes, several ambassadors experienced conditions constraining the translation of their learning outcomes into clinical practice. To improve the efficacy of the training programme, continuous refresher courses should be added, while enhanced support at organisational and political levels is necessary for SDM to become an integral feature of the clinical encounter.

**Trial registration:**

Not applicable.

**Supplementary Information:**

The online version contains supplementary material available at 10.1186/s12913-022-07639-6.

## Background

Shared decision-making (SDM) is a cornerstone of patient-centred healthcare and is increasingly highlighted as an ideal model for making health-related decisions in encounters between medical doctors (MDs) and their patients [1, 2]. SDM is referred to as a partnership between MD and patient, where the patient is presented with available treatment options based on existing evidence and is informed about the differences between them, including advantages and disadvantages [3]. The MD and the patient then make decisions together, choosing the option best suited for the patient’s preferences and life circumstances [3, 4].

Several rationales for implementing SDM in healthcare services have been highlighted in the literature. In particular, there is an ethical rationale for SDM, which argues that SDM ensures respect for the individual’s autonomy, and justice, by ensuring that the patient’s preferences and wishes are emphasised in the care and treatment they receive [3, 4]. Other rationales include that SDM may increase satisfaction among patients [5–7], improve the working environment for healthcare professionals [8], and ensure a more efficient use of resources [9]. Thus, SDM is perceived as an approach to address some of the challenges that healthcare systems all over the world are facing, including ageing populations and an increasing number of people living with one or more chronic illnesses.

In spite of these rationales and the broad interest in SDM, it is not routine practice in healthcare, either in Denmark or internationally [2, 10, 11]. Conditions related to the structure of healthcare systems and to patients and MDs have a great impact on the extent to which SDM is able to develop in clinical practice [12]. Several studies have suggested that enhancing healthcare professionals’ SDM knowledge and skills through training is important for implementing SDM in healthcare [13, 14]. The number of SDM training programmes for healthcare professionals has therefore increased rapidly worldwide; however, evidence about the effects of these training programmes is sparse, as only a few have been evaluated, and of these even fewer evaluations have been published [15]. Among the training programmes targeting MDs that have been evaluated, no clear effect on knowledge and skills has been shown [16]. Furthermore, published evaluations of SDM training programmes are difficult to compare, due to differences in evaluation design and strategies [10].

In Denmark, the trade union ‘Junior Doctors Denmark’ developed an SDM training programme in 2016, called the ‘SDM Ambassador course’, training junior doctors to become ambassadors in SDM. The aim of the course is to provide the ambassadors with the knowledge and skills to use SDM in clinical encounters with patients, and to disseminate SDM among colleagues, thereby enhancing the use of SDM in the Danish healthcare system. This study represents an external and independent evaluation of the SDM Ambassador course conducted by a university research group.

The objective of this mixed methods study is to evaluate the SDM Ambassador course, focusing on satisfaction with learning outcomes among the SDM ambassadors, and their experiences with using SDM with patients and disseminating it among colleagues. To achieve this the following questions will be addressed in this study:

1. To what extent are the ambassadors satisfied with their learning outcomes from the SDM Ambassador course, and how do they describe their satisfaction with the learning outcomes?
2. To what extent do the ambassadors use SDM with patients in clinical practice, and how do they experience the SDM that does take place?
3. To what extent do the ambassadors disseminate SDM among colleagues, and how do they experience the dissemination of SDM that does take place among colleagues?

## Methods

### Aim, design and setting of the study

**Fig. 1:**
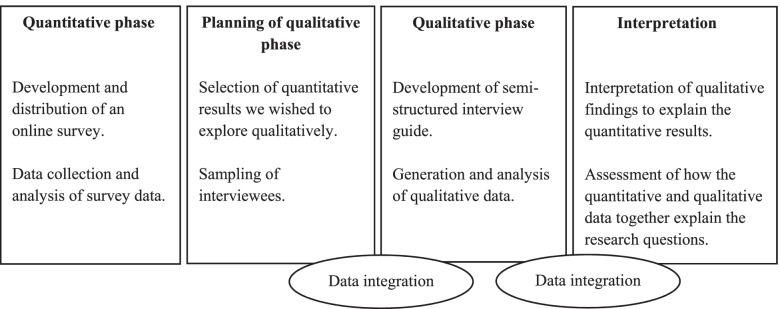
Study design and process

We conducted this study with an explanatory sequential mixed methods design consisting of two phases with the aim of evaluating the SDM Ambassador course [17]. The first phase consisted of collecting and analysing quantitative data from an online survey, followed by a second phase in which qualitative data from semi-structured interviews was generated and analysed (Fig. 1). The quantitative data was collected from 5th October to 15th November 2020. At the end of the survey respondents were able to indicate whether they wanted to participate in a follow-up interview. The generation of the qualitative data occurred from 18th November to 27th November 2020.

The SDM Ambassador course is a voluntary programme that was established in May 2016 by Junior Doctors Denmark as an offer to their members across all medical specialities. To participate in the SDM Ambassador course doctors need to be a member of the association and promise to teach SDM to their colleagues. The SDM Ambassador course consists of two days of SDM training, with only the first day of training being obligatory. The training focuses on both the theoretical and practical aspects of SDM, including communication skills and the use of decision aids.

### Characteristics of participants

Both former and active ambassadors, trained between May 2016 and September 2020, were invited to participate in the study. This resulted in a total of 185 ambassadors (55 former and 130 active) receiving an online survey. In total, 29 wished to participate in an interview, of whom we selected 10 ambassadors by a maximum variation sampling strategy, to best reflect a diverse group. This meant that we sampled interviewees who differed with regard to their answers to questionnaire items concerning learning outcomes, and usefulness and dissemination of SDM, as well as with regard to their gender, medical speciality and the number of training days they had attended.

### Data Collection

**Fig. 2:**
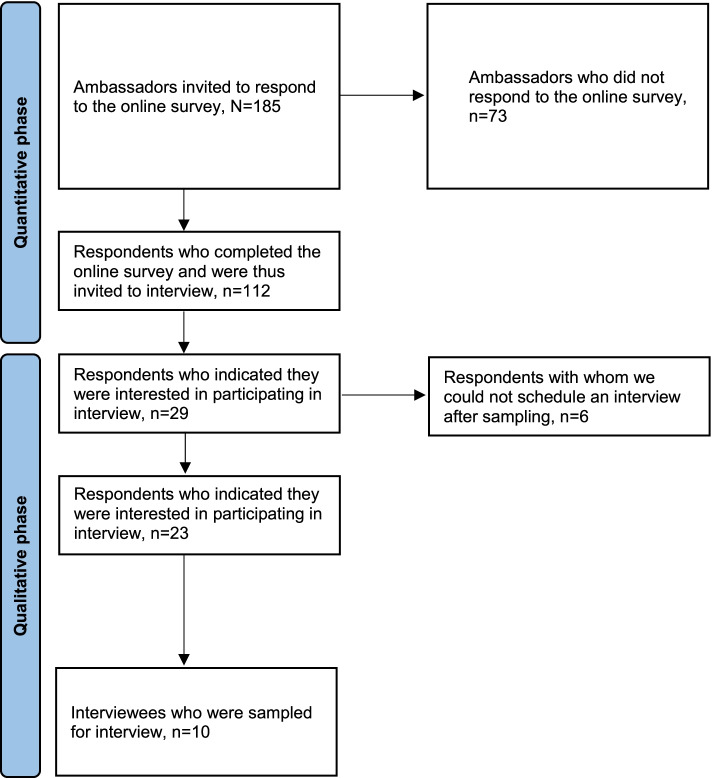
Flowchart of the study population and sampling of interviewees

The ambassadors’ names and email addresses were obtained via Junior Doctors Denmark’s membership system, with consent from the ambassadors themselves. An invitation was sent to the 185 ambassadors by an email including a link to the online survey. Two weeks after the distribution of the online survey, a reminder was sent out, with a second reminder after a further one week interval. After the second reminder, the response rate was approximately 30% only. The ambassadors who had not completed the survey were then contacted by telephone, or were sent a text message that encouraged them to participate if they had not answered the telephone call. In total, 112 ambassadors responded to the survey, corresponding to a response rate of 61%, out of which 10 ambassadors participated in interviews. The interviews were conducted and recorded over Zoom (n=6), telephone (n=3), or physically at the university campus (n=1); and lasted between 25 and 60 min. Figure 2 presents a flow diagram of the participants in this study.

The online survey (see Additional file 1) was developed in SurveyXact and included information about the evaluation and its’ purpose, data processing and storage, and also a declaration of consent form. The survey was initiated with items relating to the demographic characteristics of the ambassadors (five items) as well as items about their participation in the SDM Ambassador course (seven items). This study reports on the findings related to items concerning the ambassadors’ satisfaction with their learning outcome, and their use and dissemination of SDM in clinical practice. Satisfaction with the learning outcome was measured by three items, based on whether the ambassadors were satisfied with their SDM knowledge, competencies, and communication skills, respectively. This was assessed by five response categories, from ‘strongly disagree’ to ‘strongly agree’. The usefulness and dissemination of SDM was measured by asking the respondents whether they had used SDM knowledge and competencies gained from the Ambassador course in their clinical practice, or disseminated their SDM knowledge and competencies in their clinical context. Respondents were able to answer ‘Yes/No’ and ‘Do not know’. If they replied ‘No’, they were asked a question about the reason why they had not used or disseminated SDM, with predefined response categories and a free text field. For dissemination of SDM, there was an additional item for the ambassadors who stated that they had disseminated SDM, concerning how they had disseminated it, for which there were predefined response categories and a free text field. The content validity of the questionnaire was assessed by a pre-test with a steering committee from Junior Doctors Denmark, with in-depth knowledge about the medical profession and the Ambassador course (n=3), and a pilot test with persons from the target group (n=4).

 The subsequent semi-structured interviews were based on a standard interview guide, which was adapted to the individual interviewee’s questionnaire responses. The standard interview guide contained an introduction and the themes investigated in this study: implications for practice, including satisfaction with learning outcomes, usefulness of SDM, and dissemination of SDM. Each theme included a representation of the interviewee’s questionnaire responses, which they were then asked to elaborate. For example, interviewees who in the questionnaire responded that they had used SDM in their clinical practice were asked to describe how they used SDM in patient encounters. Interviewees who had responded that they had not used SDM in clinical practice were asked to elaborate on the reasons given in the questionnaire (Additional file 1). Every interviewee was asked to describe his/her own perception of what SDM is, and the usefulness and dissemination of SDM, in order to provide a deeper understanding of the underlying meaning of the interviewees’ questionnaire responses. Thus, the interview guide served as a guideline for exploring the pre-selected quantitative results, while also providing the possibility for the interviewees to narrate new aspects in their responses. The interview guide was discussed at a steering committee meeting and a pilot test was conducted with an ambassador from Junior Doctors Denmark.

### Analysis

Survey data was retrieved from SurveyXact to SPSS (version 27), where the data was filtered and recoded. Descriptive statistics were followed by χ^2^-tests to examine the correlation between the demographic variables and satisfaction with learning outcomes, usefulness, and the dissemination of SDM, respectively. The processing of the quantitative data formed the basis for the planning of the qualitative phase, including sampling, interview guide and analytical focus. All qualitative interviews were transcribed, and the qualitative data was imported into NVivo (version 20.3.2). The individual interviews were encoded independently by two researchers. A thematic network analysis was conducted, based on the model presented by Attride-Stirling (2001) [18]. After processing both survey and interview data, we integrated the results.

## Results

The typical participant was a woman aged 35, in specialist training to be a general practitioner, who had had become an ambassador in 2018, participated in the first day of training only, and was still a part of the ambassador programme (Table 1).

**Table 1:** Characteristics of the study population

**Sex**	**n (%)**
Female	94 (84)
Male	18 (16)
**Age**	**n (%)**
≤ 29	10 (9)
30-34	61 (54)
35-39	33 (22)
≥ 40	17 (15)
Mean	35
**Employer**^**a**^	**n (%)**
North Denmark Region	6 (5)
Central Denmark Region	26 (23)
Region of Southern Denmark	24 (21)
Region Zealand	10 (9)
Capital Region of Denmark	41 (37)
General practice	8 (7)
Central government (universities, government agencies, etc.)	3 (3)
Unemployed	1 (1)
Other	2 (2)
**Level of education**	**n (%)**
Internship	3 (3)
Introductory position	28 (25)
Specialist training	53 (47)
Medical specialist	7 (6)
Clinical assistant/research position	10 (9)
Unclassified position	9 (8)
Other	2 (2)
**Medical speciality**	**n (%)**
General medicine	31 (28)
Medical specialities	27 (24)
Surgical specialities	21 (19)
Internal medical specialities	14 (13)
Paraclinical specialities	8 (7)
Emergency medicine	7 (6)
Other	3 (3)
**Year of enrolment in the Ambassador course**^**b**^	**n (%)**
2016	22 (20)
2017	17 (15)
2018	32 (29)
2019	24 (21)
2020	17 (15)
**Days of SDM training completed**	**n (%)**
First day of SDM training	76 (68)
First and second day of SDM training	36 (32)
**Enrolled in the Ambassador course at time of data collection**	**n (%)**
Yes	75 (67)
No	17 (15)
Unknown	20 (18)
**Total**	**N (%)** 112 (100)

^a^ Multiple response allowed

^b^ Corresponding to year of completion of the first day of SDM training

### Satisfaction with learning outcomes

Most of the survey respondents were satisfied with their SDM learning outcomes from the ambassador course: knowledge (73%), competencies (57%), and communication skills (66%). Thus, fewer respondents were satisfied with their competencies within SDM than with their knowledge and communication skills. Several respondents indicated that they were neither satisfied nor dissatisfied with their SDM learning outcomes: knowledge (17%), competencies (29%), and communication skills (25%).

Associations between the respondents’ satisfaction with their learning outcomes and their use and dissemination of SDM with patients and among colleagues are shown in Table 2. Neither of the chi-square tests were significant (Table 2).

**Table 2:** Associations between the respondents’ satisfaction with learning outcomes and use and dissemination of SDM

**Use of SDM with patients in clinical practice**	Used SDM, n (%)	Did not use SDM, n (%)	Unknown, n (%)
**Satisfaction with SDM knowledge, p=0.27**			
Satisfied	67 (76)	11 (61)	4 (67)
Neither satisfied nor dissatisfied	14 (16)	3 (16)	2 (33)
Dissatisfied	7 (8)	4 (22)	0 (0)
Total	88 (100)	18 (100)	6 (100)
**Satisfaction with SDM competencies, p=0.20**			
Satisfied	54 (61)	6 (33)	4 (67)
Neither satisfied nor dissatisfied	24 (27)	7 (39)	1 (16)
Dissatisfied	10 (12)	5 (28)	1 (16)
Total	88 (100)	18 (100)	6 (100)
**Satisfaction with SDM communication skills, p=0.28**			
Satisfied	60 (68)	10 (56)	4 (67)
Neither satisfied nor dissatisfied	22 (25)	4 (22)	2 (33)
Dissatisfied	6 (7)	4 (22)	0 (0)
Total	88 (100)	18 (100)	6 (100)
**Dissemination of SDM among colleagues**	**Disseminated** **SDM, n (%)**	**Not disseminated SDM, n (%)**	**Unknown, n (%)**
**Satisfaction with SDM knowledge, p=0.50**			
Satisfied	52 (79)	28 (65)	2 (67)
Neither satisfied nor dissatisfied	9 (14)	9 (21)	1 (33)
Dissatisfied	5 (7)	6 (14)	0 (0)
Total	66 (100)	43 (100)	3 (100)
**Satisfaction with SDM competencies, p=0.42**			
Satisfied	42 (64)	20 (47)	2 (67)
Neither satisfied nor dissatisfied	15 (23)	16 (37)	1 (33)
Dissatisfied	9 (13)	7 (16)	0 (0)
Total	66 (100)	43 (100)	3 (100)
**Satisfaction with SDM communication skills, p=0.10**			
Satisfied	49 (74)	24 (56)	1 (33)
Neither satisfied nor dissatisfied	11 (17)	15 (35)	2 (67)
Dissatisfied	6 (9)	4 (9)	0 (0)
Total	66 (100)	43 (100)	3 (100)

In the qualitative interviews, the ambassadors said that they had gained more in-depth knowledge about involving patients in decision-making, and that their interaction with their patients had improved. Yet several ambassadors found it difficult to assess their satisfaction with their SDM learning outcomes. They perceived SDM as an abstract concept and felt that their SDM skills could be further improved. This might be the reason why a relatively large proportion of respondents in the survey indicated that they were neither satisfied nor dissatisfied with their SDM learning outcomes. Several ambassadors felt that they had sufficient knowledge about SDM but that it was difficult to translate this knowledge into clinical practice. Table 3 shows the integration between the quantitative and qualitative data regarding the ambassadors’ learning outcomes.

**Table 3:** Data integration of results concerning learning outcomes

**Quantitative results**	**Qualitative findings**	**Data integration**
**Learning outcomes** *Knowledge* 73% satisfied 17% neither satisfied nor dissatisfied 10% dissatisfied *Competencies* 57 % satisfied 29 % neither satisfied nor dissatisfied 14 % dissatisfied *Communication skills* 66 % satisfied 25 % neither satisfied nor dissatisfied 9 % dissatisfied	*‘… I feel that I have a good contact with my patients – it gives me more comfort.’* *‘… I can certainly improve.’*	Satisfaction with learning outcomes is due to awareness of patient involvement in the clinical encounter as well as to the experience of a better interaction with the patients. SDM is a concept which is complex and to which ambassadors find it hard to relate. The ambassadors struggle with assessing their learning outcomes due to the lack of a basis for comparison. However, they feel that they can always improve their skills.

### Usefulness of shared decision-making

In the survey, a majority of respondents (79%) reported that they had used SDM with patients in clinical practice (Table 3). Among ambassadors who had not used SDM (18 survey respondents) the main reasons for not using SDM were lack of SDM knowledge, tools, and competencies (39%), the perception that it was difficult to introduce new ways of working as a new employee in an established workplace (28%), and lack of time (22%); with the option of selecting more than one reason. In the interviews, the ambassadors explained that SDM could be used as both a mindset and a method. Several of the ambassadors emphasised that they mostly used SDM as a mindset in their encounter with patients. They found it more difficult to use SDM as a method, partly because they felt that they did not have the necessary SDM tools, such as decision aids. In the interviews, several contextual factors in the ambassadors’ clinical practice were emphasised as being decisive for whether and how the ambassadors translated their learning outcomes into use in clinical practice. These included time constraints, their medical speciality, and their professional experience and medical knowledge. Key points from the quantitative and qualitative results for the usefulness of SDM and the data integration are shown in Table 4.

**Table 4:** Data integration of results concerning the usefulness of SDM

Quantitative results	Qualitative findings	Data integration
**Use of SDM** 79% have used SDM 16% have not used SDM 5% do not know	*‘… I carry the mindset in all my consultations…’* *‘As a new doctor one will always think it is a challenge to provide guidance on a treatment you have not had a lot of experience with.’*	Use of SDM by the ambassadors is felt to be easier as a mindset than as a method. Personal characteristics of the doctor such as learning outcome and professional experience, as well as contextual conditions such as medical specialities and limited time, shape the ambassadors’ use of SDM in the clinical encounter.

### Dissemination of shared decision-making

More than half of the respondents (59%) in the survey stated that they had disseminated SDM among colleagues (Table 3). 85% of the ambassadors indicated that they had told one or more colleagues about SDM and 67% had made presentations about SDM to their medical department. The main reasons for not disseminating SDM were issues related to the ambassadors’ work practices, including competing work tasks (51% out of 43), a belief that SDM did not fit into their clinical practice (26%), and a feeling of insufficient knowledge and competencies to communicate about SDM (23%). The ambassadors emphasised that they primarily disseminated SDM by telling colleagues about SDM and by teaching SDM in their medical departments. In particular, the interviewed ambassadors emphasised that they did not feel sufficiently equipped to disseminate SDM among their colleagues, as it was challenging to teach others something that they did not feel comfortable in using in practice themselves. This could explain why fewer respondents in the survey stated that they had disseminated SDM among colleagues than used SDM with patients in clinical practice. In the interviews, several ambassadors emphasised that they needed to be part of a network with other ambassadors to exchange ideas and discuss issues, for example by preparing and making presentations together. The ambassadors perceived various contextual factors in the clinical practice as being important for their dissemination of SDM. These included scheduled teaching sessions specifically to facilitate dissemination of SDM. However, the experience that presentations for colleagues were not sufficient to disseminate SDM, along with comments (especially from older colleagues) that ‘we are already practicing SDM’, were perceived as barriers. Key points from the quantitative and qualitative results for dissemination of SDM and the data integration are shown in Table 5.

**Table 5:** Data integration of results concerning the dissemination of SDM

Quantitative results	Qualitative findings	Data integration
**Dissemination of SDM** 59% have disseminated SDM 38% have not disseminated SDM 3% do not know	*‘… I do not think I have found a good way to implement SDM in my everyday practice which has kept me from disseminating the message.’* [about making a presentation among colleagues] ‘*…Well, that was interesting and then nothing happened.’*	Personal characteristics of the doctor, such as the fact that their learning outcome does not equip them adequately to disseminate SDM, and contextual conditions, such as integrated teaching and cultural norms at the clinical department, shape the ambassadors’ dissemination of SDM in the clinical encounter. SDM presentations are not sufficient to promote dissemination of SDM in the Danish healthcare system.

## Discussion

### Main findings

This study used mixed methods to evaluate junior doctors’ satisfaction with their learning outcomes, as well as their use and dissemination of SDM among patients and colleagues, respectively, after participating in the SDM Ambassador course. In general, the ambassadors were satisfied with their learning outcomes, and a majority of the ambassadors indicated that they had used and disseminated SDM in their clinical practice. The context of the ambassadors’ clinical practice was significant for their experience of usefulness and dissemination of SDM.

### Comparison with the literature

The ambassadors’ satisfaction with their learning outcomes from the Ambassador course is in line with the international literature, which shows that even short-term SDM training programmes have a positive effect on MDs’ learning outcomes [13, 19–22]. However, our results are not strictly comparable with the results of these studies as they were primarily collected from MDs within one medical specialism. Thus, our study contributes to a broader picture of the effects of training programmes by focusing on MDs’ satisfaction with their learning outcomes within different medical specialities. Also, our results showed that fewer ambassadors were satisfied with their competencies compared with their knowledge and communication skills within SDM. This finding was explained and nuanced in our interviews, in which several ambassadors said that it was difficult for them to apply their learning outcomes in their clinical practice, although they did have sufficient knowledge and materials about SDM. This indicates that short-term training programmes in SDM are not sufficient if MDs are to be equipped to use and disseminate SDM routinely in their clinical practice with patients and among colleagues. Thus, even though SDM training is highly valued and considered important among MDs, this finding indicates that SDM skills are of most significance when training also leads to a change in mindset within a supportive context.

This study showed that most of the ambassadors used (79%) and disseminated (59%) SDM following the Ambassador course. To our knowledge, only a few studies have examined whether and how MDs use and disseminate SDM, with patients and among colleagues respectively, after an SDM training programme. Studies have shown varied results which can be attributed to differences in study designs and methods of measurement [19, 22–24]. Unlike our study, Körner et al. (2012) found that daily interaction with colleagues in relation to a train-the-trainer programme in SDM, for providers in executive positions, affected the number of healthcare professionals using SDM in clinical practice [23]. Our results also indicate that there is not one shared understanding of what SDM is among MDs. This finding is in accordance with Makoul and Clayman’s (2006) review which concludes that there is no overall shared definition of SDM to be found in the literature in the context of physician-patient encounters [25]. According to the authors, the lack of a shared definition of SDM can limit the productivity of research on SDM as it can lead to inconsistency in the measurement of SDM and make comparisons across studies difficult [25]. Furthermore, it is likely that the ambassadors are not aware of the fact that dissemination of SDM is a practice that can be carried out unintentionally when ambassadors use SDM themselves, as they may inspire their colleagues to adapt this mindset. Overall, SDM is an ambiguous concept, inherent in complex clinical practices, that relates both to approach and behaviour. This complexity makes it difficult for MDs, and researchers, to self-assess the learning outcomes, usefulness, and dissemination of SDM following a training programme.

Different conditions in the clinical context shaped the ambassadors’ use and dissemination of SDM, including lack of time, complexity in the clinical situation, the medical hierarchy, ethical dimensions, and a lack of clinical experience. These contextual conditions are known barriers in the literature [12, 19, 22–24, 26–28]. Thus, this study does make a contribution by confirming that these conditions also influence the ambassadors’ usefulness and dissemination of SDM in the context of the Danish healthcare system. A short SDM training programme cannot alter these contextual conditions.

Furthermore, based on our results, a more critical appraisal of the weight given to SDM training and practice is warranted. Complexity in clinical encounters, and diversity in patient preferences and needs, treatment and care situations, highlight the importance of carefully reflecting upon when SDM is warranted, and when the overall approach of patient-centeredness is more appropriate for the context of the individual patient. Evidence points to the fact that patients prefer to be well informed, but that their preferences for SDM are ambiguous [29–31]. This should be reflected in training programmes and approaches to implementing SDM into routine practice among MDs. Thus, our findings point to the importance of recognising the complex cultural and structural conditions that may act as barriers to SDM. SDM is not straightforward, and we need to know more about when, how (and how much) and with whom SDM is appropriate.

### Strengths and limitations

This study should be considered within the context of its methodological strengths and limitations. We used mixed methods to carry out an in-depth evaluation of the Ambassador course. Our quantitative results provided a broad knowledge of the ambassadors’ satisfaction with their learning outcomes, as well as the extent to which the ambassadors experienced using and disseminating SDM in their clinical practice. These results were nuanced and contextualised by the interviews, which drew attention to several contextual conditions that shaped the ambassadors’ perception of their learning outcomes, as well as their experiences with using and disseminating SDM. This comprehensive knowledge, gained from triangulating quantitative and qualitative data, underlines the strength of our mixed methods design.

Our data collection approach led to a relatively high response rate (61%), which reduced the risk of selection bias in our results. However, the Ambassador course is a voluntary training programme, from which it can be assumed that the ambassadors in general had a more positive attitude towards SDM and the training programme. Thus, our results cannot be generalised to every member of Junior Doctors Denmark. Furthermore, our use of self-reported measures of learning outcomes, usefulness, and dissemination of SDM might have an implication in that MDs may be limited in their ability to evaluate their own standards [32]. This is important to keep in mind when evaluating the results. In addition, the cross-sectional design does not permit an assessment of causality within our results. It is likely that the ambassadors’ satisfaction with their learning outcomes, use, and dissemination of SDM were not solely a result of their participation in the Ambassador course. However, we consider our use of maximum variation sampling as a strength, regarding the study’s applicability. By ensuring as much variation as possible among the interviewed ambassadors, in terms of their learning outcomes, use and dissemination of SDM, we have achieved a more representative evaluation of the Ambassador course than if we had only interviewed those who, for example, were the most satisfied with the ambassador course.

Our study focuses on JMDs’ experiences with SDM training. Future studies should therefore investigate whether the patients feel more included in health-related decisions and experience better treatment because of SDM training programmes. In the long run interventions and evaluations should target multiple health professionals, including MDs, as well as patients and their relatives. However, the interventions need to take place in a context where the cultural and structural conditions that work as barriers for SDM are addressed. Thus, interventions need to be accompanied by organisational and political support so that the clinical context facilitates both the use and dissemination of SDM.

### Future perspectives

Based on this study’s results, future short-term SDM training programmes should supplement SDM teaching with regular refresher courses that can be taken online, to accommodate MDs’ busy and changeable professional and everyday lives. Furthermore, it is relevant to discuss whether it is appropriate for relatively young and newly-qualified MDs to be agents of change in the Ambassador course, as this study has shown that this group of MDs lacks impact in their clinical environments, especially among their older and more experienced colleagues. Thus, future SDM programmes should focus on targeting MDs with different levels of medical experience and impact in their clinical environments. Following on from this, it would be beneficial if SDM was introduced at medical schools so that future MDs would be introduced to SDM earlier in their medical careers. Thus, SDM education would not be limited to those who participate in a voluntary SDM training programme. In addition, an interdisciplinary effort is required if SDM training programmes such as the Ambassador course are to fulfil their potential. This is because a patient’s pathway involves contact with various health professions in the healthcare system, not only MDs. Therefore, it will be appropriate that healthcare providers in executive positions introduce SDM training programmes to their entire departments, based on their local structures and contact with patients.

Finally, there is a need for a clear definition of SDM and a better understanding among MDs, as well as other healthcare professionals engaging with patients, of how and when it is needed in patient encounters if SDM as both a skill and a mindset is to be implemented routinely in MDs’ clinical practice.

## Conclusions

Despite overall satisfaction with their learning outcomes, several ambassadors found it difficult to translate their learning outcomes into use and dissemination of SDM in their clinical practice. Thus, the programme can be improved to obtain its full potential, for example by continuous refresher courses. In addition, this study highlights the fact that action needs to be taken both at an organisational and political level for SDM to become an integral part of the clinical encounter.

## Supplementary Information

**Additional file 1. ** Online survey. Online survey/questionnaire used in this study.

## Data Availability

The data generated and analysed during the current study are available from the corresponding author on reasonable request.

## Abbreviations

SDM: Shared decision-making.
Junior Doctors Denmark: Danish Association of Junior Doctors in Denmark
JMDs: Junior medical doctors
MDs: Medical doctors
