# Supplementary material for: Evaluation of a national programme to improve shared decision-making skills among junior medical doctors in Denmark: a mixed methods study of satisfaction, usefulness, and dissemination of learning outcomes in clinical practice
Source: BMC Health Serv Res. 2022 Feb 23;22:245. doi: 10.1186/s12913-022-07639-6 (PMC8867664; doi:10.1186/s12913-022-07639-6)
Supplement: Supplementary file 1 — Additional file 1. Online survey. Online survey/questionnaire used in this study. [file 12913_2022_7639_MOESM1_ESM.docx]

**Additional file 1: Online Survey**

**Do you want to help us improve the shared decision-making Ambassador course?**

| We are evaluating the shared decision-making Ambassador course run by Junior Doctors Denmark, to generate knowledge that can improve the course.  Feedback on your experiences of the Ambassador course are crucial to ensure that it continues to offer the best preparation to promote and disseminate the use of shared decision-making – even though you are not an active ambassador anymore.   **The survey takes 10 to 15 minutes to complete**. Your answers will be submitted when you click ’Finish’ at the end of the survey.    Thank you so much for your time! We hope you will help us by answering this survey before 15^th^ November 2020.  **Declaration of consent** |
| --- |

It is voluntary to participate in the survey.

| You can withdraw your consent at any time by contacting the research group leader, by email or telephone.  The information collected will be processed confidentially in accordance with the purpose of the project. It is only the research group who will have access to data.  The results from the survey will be used in a peer-reviewed article, an evaluation report, and a Master thesis. The data will be published in such a manner that your answers cannot be attributed to you individually.   We ask you for permission to collect and process survey data. You are giving your consent and agreement to participate in the survey by clicking ‘**Next**’.  **Background Information** |
| --- |

The following questions deal with your background information.

|  |
| --- |

1. What is your gender?

(1) ❑ Female

(2) ❑ Male

(3) ❑ Other _____

2. Which year where you born?

_____

3. Who is your employer?

(It is possible to select more than one category)

(1) ❑ The region of northern Jutland

(2) ❑ Central Jutland region

(3) ❑ South Denmark region

(4) ❑ Region Zealand

(5) ❑ The Capital region

(8) ❑ General practice

(7) ❑ The state (university, board etc.)

(6) ❑ I am not working

(9) ❑ Other

4. What stage have you reached in your medical training?

(1) ❑ Internship

(2) ❑ Introductory position

(3) ❑ Specialist training

(4) ❑ Medical specialist

(5) ❑ Clinical assistant/research position

(6) ❑ Unclassified position

(7) ❑ Other

5. In which medical speciality is your current employment?

_____

| **Your participation in the shared decision-making Ambassador course**  In this survey you are defined as a shared decision-making ambassador if you have participated in at least one training day.  In 2019 it became possible for ambassadors to participate in an extra training day (training day 2) which digs deeper into the methods of shared decision-making. |
| --- |

6. How did you hear about the Ambassador course?

(You can choose more than one response category)

(1) ❑ Through a colleague

(2) ❑ Through management

(3) ❑ Through Junior Doctors Denmark (newsletters, social media, websites, etc.)

(4) ❑ Through other social media

(5) ❑ Through another ambassador

(6) ❑ Another way_____

7. When did you become an ambassador?

(1) ❑ 2016

(2) ❑ 2017

(3) ❑ 2018

(4) ❑ 2019

(5) ❑ 2020

8. Which training days have you completed?

(1) ❑ Training day 1

(2) ❑ Training days 1 and 2

9. Why did you choose to become an ambassador?

(You can choose more than one response category)

(1) ❑ In order to gain knowledge and tools to involve patients and their relatives

(2) ❑ Because it is a politically-prioritised subject

(3) ❑ To give my patients the best treatment

(4) ❑ Because people in my professional environment, e.g. a manager or a colleague, encouraged me to participate

(5) ❑ To become part of a network with knowledge-sharing

(6) ❑ To accommodate expectations from patients and their relatives

(7) ❑ To optimise my professional profile, e.g. in relation to a future medical position

(8) ❑ Other reasons_____

10. Are you still an active ambassador?

(1) ❑ Yes

(2) ❑ No

(3) ❑ Do not know

10.b. Why are you no longer an active ambassador?

(You can choose more than one response category)

(5) ❑ I do not have time

(2) ❑ It is no longer relevant to me

(1) ❑ I am no longer a member of Junior Doctors Denmark

(3) ❑ I am no longer interested in member offers, newsletters, etc.

(4) ❑ Another reason_____

| **Learning outcome**  The following questions are about your satisfaction with your learning outcome from the training day(s).  If you have participated in both training days 1 and 2, please assess your overall impression of the two days. |
| --- |

11. How satisfied were you with the following?

|  | Very satisfied | Satisfied | Neither satisfied nor dissatisfied | Dissatisfied | Very dissatisfied |
| --- | --- | --- | --- | --- | --- |
| The teachers | (1) ❑ | (2) ❑ | (3) ❑ | (4) ❑ | (5) ❑ |
| The course content | (1) ❑ | (2) ❑ | (3) ❑ | (4) ❑ | (5) ❑ |
| The organisation of the teaching | (1) ❑ | (2) ❑ | (3) ❑ | (4) ❑ | (5) ❑ |
| The length of the training day(s) | (1) ❑ | (2) ❑ | (3) ❑ | (4) ❑ | (5) ❑ |

12. In your work as a doctor today, how much do you agree with the following statements?

|  | Strongly agree | Agree | Neither agree or disagree | Disagree | Strongly disagree |
| --- | --- | --- | --- | --- | --- |
| I am satisfied with my knowledge about shared decision-making | (2) ❑ | (3) ❑ | (4) ❑ | (5) ❑ | (6) ❑ |
| I am satisfied with my competencies within shared decision-making | (2) ❑ | (3) ❑ | (4) ❑ | (5) ❑ | (6) ❑ |
| I am satisfied with my communication skills within shared decision-making | (2) ❑ | (3) ❑ | (4) ❑ | (5) ❑ | (6) ❑ |
| I am interested in learning more about concepts within shared decision-making | (2) ❑ | (3) ❑ | (4) ❑ | (5) ❑ | (6) ❑ |

| **Usefulness of shared decision-making in clinical practice**  The following questions are about your use of the knowledge and competencies you have acquired through the Ambassador course. |
| --- |

13. Have you used knowledge and competencies from the Ambassador course in your clinical practice?

(1) ❑ Yes

(2) ❑ No

(3) ❑ Do not know

13.b. Why have you not used knowledge and competencies from the Ambassador course in your clinical practice?

(Feel free to select more than one response category)

(2) ❑ I lack time

(1) ❑ I lack support from management

(4) ❑ It does not match the clinical guidelines in my clinical practice

(10) ❑ I lack knowledge, tools, and competencies for shared decision-making

(3) ❑ It is hard to introduce new ways of thinking and working in an established workplace

(9) ❑ Another reason_____

14. Have you disseminated knowledge and methods about shared decision-making in your clinical practice?

(1) ❑ Yes

(2) ❑ No

(3) ❑ Do not know

14.a. How have you disseminated knowledge and methods about shared decision-making?

(You can choose more than one response category)

(1) ❑ I have told one or more colleagues about shared decision-making

(2) ❑ I have held presentations about shared decision-making in my medical department

(8) ❑ I have held presentations about shared decision-making outside my medical department

(3) ❑ I have written posts about shared decision-making

(4) ❑ Via social media, e.g. LinkedIn, Facebook, Twitter, blog or podcast

(9) ❑ By participating in research

(6) ❑ Via journal-clubs

(7) ❑ Another method_____

14.b. Why have you not disseminated knowledge and methods about shared decision-making?

(Feel free to select more than one response category)

(6) ❑ I have competing work tasks

(1) ❑ I lack support from management

(2) ❑ I experience lack of interest from my colleagues

(5) ❑ I am no longer interested in shared decision-making

(3) ❑ I lack knowledge and competencies to be able to disseminate

(4) ❑ It does not fit into the workflows in my clinical practice

(7) ❑ Another reason_____

| **Networking opportunities within the Ambassador course**  Junior Doctors Denmark offer several networking opportunities as part of the Ambassador course.  The following questions are about your use of these networking opportunities. |
| --- |

15. Have you used networking opportunities within the Ambassador course?

(You can choose more than one response category)

(4) ❑ Yes, I have contact with other ambassadors

(2) ❑ Yes, I have read newsletters from Junior Doctors Denmark

(3) ❑ Yes, I have participated in conferences related to shared decision-making

(1) ❑ Yes, I am a member of the closed Facebook group ’Shared decision-making Ambassadors’

(5) ❑ No

(6) ❑ Do not know

16. Do you have any suggestions for other opportunities for networking and knowledge-sharing within the Ambassador course?

________________________________________
________________________________________
________________________________________
________________________________________
________________________________________
________________________________________

| **Working life and career**  The following questions are about the importance of the Ambassador course for your working life and career. |
| --- |

17. Has it been important for your working life and career that you are or have been an ambassador?

(1) ❑ Yes

(2) ❑ No

(3) ❑ Do not know

17.a. How has it been important for your working life and career that you are or have been an ambassador?

(Feel free to select more than one response category)

(4) ❑ I have been given new/more work tasks related to shared decision-making

(1) ❑ It has made my everyday life as a doctor more meaningful

(8) ❑ I treat my patients better

(3) ❑ It has become a part of my professional profile

(7) ❑ It has affected my choice of medical speciality

(5) ❑ It has contributed to the fact that I have gained a position within the medical speciality that I prefer

(2) ❑ I have gained recognition, e.g. managerially, collegially, or in the form of a wage increase

(6) ❑ Something else____

| **Concluding questions** |
| --- |

18. Would you recommend the Ambassador course to others?

(1) ❑ Yes

(2) ❑ No

(3) ❑ I do not know

18.a. Name up to three reasons why others should participate in the Ambassador course:

| 1. | ________________________________________ ________________________________________ ________________________________________ ________________________________________ ________________________________________ ________________________________________ |
| --- | --- |
| 2. | ________________________________________ ________________________________________ ________________________________________ ________________________________________ ________________________________________ ________________________________________ |
| 3. | ________________________________________ ________________________________________ ________________________________________ ________________________________________ ________________________________________ ________________________________________ |

19. Do you have suggestions for improvement of the Ambassador course?

________________________________________
________________________________________
________________________________________
________________________________________
________________________________________
________________________________________

| **Help us further with improving the Ambassador course**  May we contact you for a follow-up interview about themes involved in this survey?  The interview will last around 30 minutes and we expect to hold it in **weeks 47-48** (15^th^ November to 30^th^ November).  The interview can be held at a place and time that suits you – either physically or online.  If you are happy for us to contact you, please fill out your information below: |
| --- |

Name:

_____

Email:

_____

Telephone number:

________

| **Thank you for your participation!**  If you have questions or need further information you can contact the research group leader, by email or telephone.  Remember to click ‘**Finish**’ to submit your answers! |
| --- |
